# Alteration in Endoglin-Related Angiogenesis in Refractory Cytopenia with Multilineage Dysplasia

**DOI:** 10.1371/journal.pone.0053624

**Published:** 2013-01-16

**Authors:** Mónica del Rey, Miguel Pericacho, Soraya Velasco, Eva Lumbreras, José Miguel López-Novoa, Jesús María Hernández-Rivas, Alicia Rodríguez-Barbero

**Affiliations:** 1 IBMCC, Centro de Investigación del Cáncer (CIC), Universidad de Salamanca-CSIC, Salamanca, Spain; 2 IBSAL, Instituto de Investigación Biomédica de Salamanca, Salamanca, Spain; 3 Departamento de Fisiología and Farmacología, Universidad de Salamanca, Salamanca, Spain; 4 Servicio de Hematología, Hospital Universitario de Salamanca, Salamanca, Spain; Shanghai Jiao Tong University School of Medicine, China

## Abstract

The functional mechanisms involved in angiogenesis and the potential role of endoglin (ENG), recently described as a new marker for this process, have not been explored in Myelodysplastic Syndromes (MDS). In order to gain insight in MDS angiogenesis a combined analysis in bone marrow (BM) of gene expression levels, angiogenesis-related soluble factors and functional angiogenesis-related studies was carried out. Ninety-seven MDS patients and forty-two normal BM samples were studied. The morphology of the capillary-like structures originated by two endothelial cells lines in the BM environment of patients with refractory cytopenia with multilineage dysplasia (RCMD) was different from those of the remaining MDS. In addition, the BM mononuclear cells from RCMD patients displayed over-expression of *VEGF*, *HIF* and *FN1* while they showed reduced expression of *ENG* in contrast to the normal *ENG* expression of the remaining low-risk MDS and the high expression of *ENG* in high-risk MDS subtype. Moreover, higher soluble ENG and soluble FLT-1 levels in BM microenvironment were observed in RCMD cases, which distinguished them from other individuals. Therefore, the present study suggests that the patterns of angiogenesis are different between the MDS subtypes. The differences in angiogenesis observed in RCMD patients could be related to ENG abnormalities.

## Introduction

Myelodysplastic Syndromes (MDS) are a heterogeneous group of hematopoietic malignancies, characterized by ineffective haematopoiesis, hypercellular bone marrow (BM), dysplasia of at least one lineage and cytopenias in the peripheral blood [1]. These disorders are classified according to WHO criteria, which take into account types and number of cell dysplasias, percentage of blasts and cytogenetic abnormalities [2], [3]. Moreover, based on these parameters, MDS can be divided into four prognostic categories: low, intermediate-1, intermediate-2 and high risk [4]. MDS are stem cell disorders, however, some studies have recently stressed the possibility that the BM microenvironment may play a relevant role in the pathogenesis of these diseases [5]. In addition, abnormalities in signal transduction, transcription activity, cell-cycle control, epigenetic, mitochondrial DNA and angiogenesis have been related to MDS [6].

Angiogenesis is the process by which new blood vessels are formed from pre-existing vessels and it has been associated with growth, dissemination and metastasis of solid tumours [7]. In hematological malignancies, angiogenesis develops in different way than in solid tumours [8], [9]. There are conflicting evidences regarding angiogenesis in MDS; some studies have proposed that BM microvascular density (MVD) increases with MDS progression [10], whereas others suggest an increased vascularity in the early but not the latter stages of MDS [11]. Differences between MDS subtypes could explain these conflicting results and hence the importance of the discrimination between the different entities of MDS.

Endoglin (ENG) is an integral membrane glycoprotein whose properties have made it a reliable marker of tumour angiogenesis and a prime target for anti-angiogenic therapy [12]. ENG serves as co-receptor for members of the transforming growth factor beta (TGF-β) superfamily of proteins [13] and a major evidence for the pivotal role of ENG in angiogenesis is that mice lacking *Eng* (*Eng−/−*) die from cardiovascular defects at mid gestation with major defects in yolk sac vasculature [13], [14]. *ENG* is mainly expressed in proliferating vascular endothelium and its expression increases during tumour angiogenesis and inflammation [12], [13]. Elevated expression of *ENG* correlates with the proliferation of tumour endothelial cells [15] and also in hematopoietic tumours such as multiple myeloma [16] and in hairy cell leukemia [17]. The mechanism involved in the *ENG* over-expression is probably multifactorial, being hypoxia one of the most suitable candidates. In fact, many of the pathophysiological settings where *ENG* is upregulated involve hypoxic microenvironments, as is the case of tumour angiogenesis [18]. Although ENG is a membrane protein, low levels of soluble protein (sENG) can be found in extracellular medium. The appearance of this soluble protein form is probably due to proteolytic cleavage of isoform membrane as occurs with betaglycan, which can be shed by metalloproteinase 1 [19]. sENG interferes with TFG-β signalling causing endothelial dysfunction [20]. It has been demonstrated that sENG inhibits the capilar tube formation “in vitro” and increases vascular permeability [21].

Most of the studies of angiogenesis in MDS have been focused on malignant haematopoietic cells but there is growing evidence that BM-derived endothelial cells may contribute to tumour angiogenesis [22], [23]. In addition, clonal cells may have interactions with these BM endothelial cells and the contact between endothelial cells and normal or malignant haematopoietic cells is mediated by soluble angiogenic factors of the BM microenvironment [24], [25]. Therefore the role of endothelial cells in the BM malignant microenvironment and their possible relationship with the malignant clone remains to be clarified by functional studies and not only assessed by immunohistochemistry that so far has been the most used method of analyzing angiogenic activity in MDS [26].

In order to gain insight in the mechanisms involved in angiogenesis in MDS a study of the cellular expression and the BM microenvironment levels of sENG and other angiogenic factors was carried out. The results showed marked differences in the angiogenesis in the MDS subtypes, and could open new approaches in the treatment in MDS patients.

## Design and Methods

### 1. Patients Samples

A total of 97 MDS patients and 42 age-matched controls were included in the study. Classification of MDS was performed according to the World Health Organization (WHO) criteria [3]. Twenty-nine patients were diagnosed as refractory cytopenia with multilineage dysplasia (RCMD) and forty-six had other low-risk MDS excluding RCMD: nineteen of them had a refractory anemia (RA), twenty-two had a refractory anemia with ring sideroblasts (RARS) and five patients had a 5q- syndrome. The remaining twenty-two patients had a refractory anemia with excess of blasts (RAEB: high-risk MDS) (Table S1). The study was approved by the local ethical committees “Comité Ético de Investigación Clínica, Hospital Universitario de Salamanca”. Written informed consent was obtained from each patient before they entered the study.

Mononuclear cells as well as the supernatant fluid were isolated from BM of MDS patients and controls by density gradient (Ficoll). Total RNA from cells was extracted by homogenization in TRIZOL (Invitrogen, Carlsbad, CA, USA) following the protocol supplied by manufactures, and treated with RQ1 RNAse-Free DNase (Promega, Madison, USA) to eliminate genomic DNA contamination, and finally purified with RNeasy Minikit (Qiagen, Hilden, Germany). The RNA quantity and quality was determined by Agilent 2100 Bioanalyzer (Santa Clara, CA, USA). The BM supernatant fluid was collected by centrifugation. The liquid was withdrawn carefully to avoid disturbing the cells and collected in a separate container at −80°C.

### 2. BMEC-1 Cell Culture

The immortalised cell line, BMEC-1 (Bone marrow endothelial cells), kindly donated by Dr. F.J. Candal (Centers for Disease Control and Prevention, Atlanta, Georgia) was used in our studies [27]. This cell line was generated by transfecting an early passage of primary BMEC with a vector (pSVT) encoding the large T antigen of SV40. BMEC-1 express vWF/Factor VIII and maintain a phenotype similar to that of primary cells, even at high passage number.

Cells were maintained in culture at 37°C in a humidified atmosphere of 95% air and 5% CO_2_ in Medium MCDB 131 (Invitrogen) supplemented with 15% foetal calf serum (FCS), 10 ng/ml endothelial grow factor (EGF) and 1 µg/ml hydrocortisone.

### 3. HMVEC-L Cell Culture

Lung-derived normal human microvascular endothelial cells (HMVEC-L) were purchased from Clonetics (Lonza Walkersville, MD, USA) and maintained exactly as recommended by the manufacturer. Cells were cultured with the EGM-2MV bullet kit containing endothelial cell basal medium-2 (EBM-2) and the following growth supplements: hEGF, hydrocortisone, GA-1000, FBS, VEGF, hFGF-B, R^3^-IGI-1 and ascorbic acid. The experiments described in this study were performed on cells between three and four passages.

### 4. Cell Proliferation Assay

Subconfluent BMEC-1 were plated in 96-well plates to a density of 5,000 cells per well. Twelve hours after plating, cells were serum starved (5% FCS) and the BM supernatant fluid from MDS patients or controls was added (1∶10 dilution). After incubation during 24 h, 48 h or 72 h, Thiazolyl Blue Tetrazolium Bromide (MTT, Sigma, Illinois, USA) was added to each well and incubated for 4 h. The formazan crystals formed from MTT by the living cells were dissolved in the lysis buffer (10% sodium dodecyl sulfate (SDS); 5% isopropanol; 0.1M HCl) for 12 h, and the formazan purple solution was detected using a Sunrise plate reader (Bio-Tek, Instruments, Winooski, USA) at 595 nm. All experiments were performed in quadruplicate.

### 5. Endothelial Cell Tube Formation Assay

Endothelial cell tube formation was assessed as previously described Jerkic et al [28]. In brief a total of 8,000 BMEC-1 per well were plated on Matrigel® precoated plates (BD Biosciences, New Jersey, USA) and cultured in medium MCDB 131 with 15% FCS. Half an hour later, the BM supernatant fluid from MDS patients or controls was added in the wells (1∶10 diluted). After seeding on Matrigel®, cells spread and aligned with each other to develop hollow, tube-like structures. Endothelial tube formations were observed each hour during seven hours of incubation and the morphological changes were photographed at 5 h using a phase contrast inverted Zeiss Microscope (Carl-Zeiss, Jena, Germany). Each experiment was performed in duplicate. As a control in one of each 5 wells just culture medium was added. The experiment was performed in the same way with HMVEC-L. Likewise, a total of 8,000 HMVEC-L per well were plated on Matrigel® precoated plates. However, in this case, it was used its appropriate culture medium as described above.

### 6. Real-Time PCR

The expression levels of endoglin *(ENG)*, vascular endothelial grow factor *(VEGF)*, hypoxia-inducible factor 1-alpha (*HIF1*) and fibronectin (*FN1*) genes were analyzed by Real-Time PCR. First-strand cDNA was generated from 1 µg of total RNA using poly-dT as primers with the M-MLV reverse transcriptase (Promega). Real-time PCR was performed in triplicate. Each 20 µl reaction contained 300 ng of cDNA, 400 nM of each primer, and 1× iQ SybrGreen Supermix (Bio-Rad, Hercules, CA, USA). Standard curves were run for each transcript to ensure exponential amplification and to rule out non-specific amplification. The expression level of the glyceraldehyde-3-phosphate dehydrogenase (*GAPDH*) gene was used to normalize differences in input cDNA. The reactions were run on an iQ5 Real-time PCR detection system (Bio-Rad, Hercules, CA, USA). The primers were designed for specific sequences and checked by BLAST algorithm [29]. Primer sequences were as follows:

*GAPDH*- forward: 5′-CAG GGC TGC TTT TAA CTC TGG TAA-3′.

*GAPDH*- reverse: 5′-GGG TGG AAT CAT ATT GGA ACA TGT A-3′.

*ENG*- forward: 5′-AGG TGC TTC TGG TCC TCA GT-3′.

*ENG*- reverse: 5′-CCA CTC AAG GAT CTG GGT CT-3′.

*VEGF*- forward: 5′-CGA AGT GGT GAA GTT CAT GG-3′.

*VEGF*- reverse: 5′-CAC AGG ATG GCT TGA AGA TG-3′.

*HIF1*- forward: 5′-GTC ACT TTG CCA GCT CAA AA-3′.

*HIF1*- reverse: 5′-ACC AAC AGG GTA GGC AGA AC-3′.

*FN1-* forward: 5′-TCA CAG CTT CTC CAA GCA TC-3′.

*FN1-* reverse: 5′-TGG CTG CAT ATG CTT TCC TA-3′.

### 7. Enzyme-linked Immunosorbent Assay

In order to assess the concentration of proangiogenic soluble factors, an enzyme-linked immunosorbent assay (ELISA) was carried out in the BM supernatant fluid from patients and controls. The levels of sENG, soluble fms-like tyrosine kinase 1 (sFLT-1) and VEGF were assessed by using commercially available kits from R&D Systems (R&D Systems, Minneapolis, USA). The whole protocol was performed following the instructions given by the manufacturer.

### 8. Statistical Analysis

The relationship between clinical or biological data and genomic characteristics was analyzed using an independent sample ANOVA test or Mann-Whitney test. All P-values reported were two-sided and statistical significance was defined as P-value <0.05. Statistical evaluation was carried out using the SPSS 15.0 statistical software. The graphics of the study show the mean ± standard error of the mean or the median of each group that was used for the comparisons in each experiment between the different entities of MDS and the range in each case.

## Results

### BM Microenvironment from MDS Patients Induces Endothelial Proliferation

**Figure 1:**
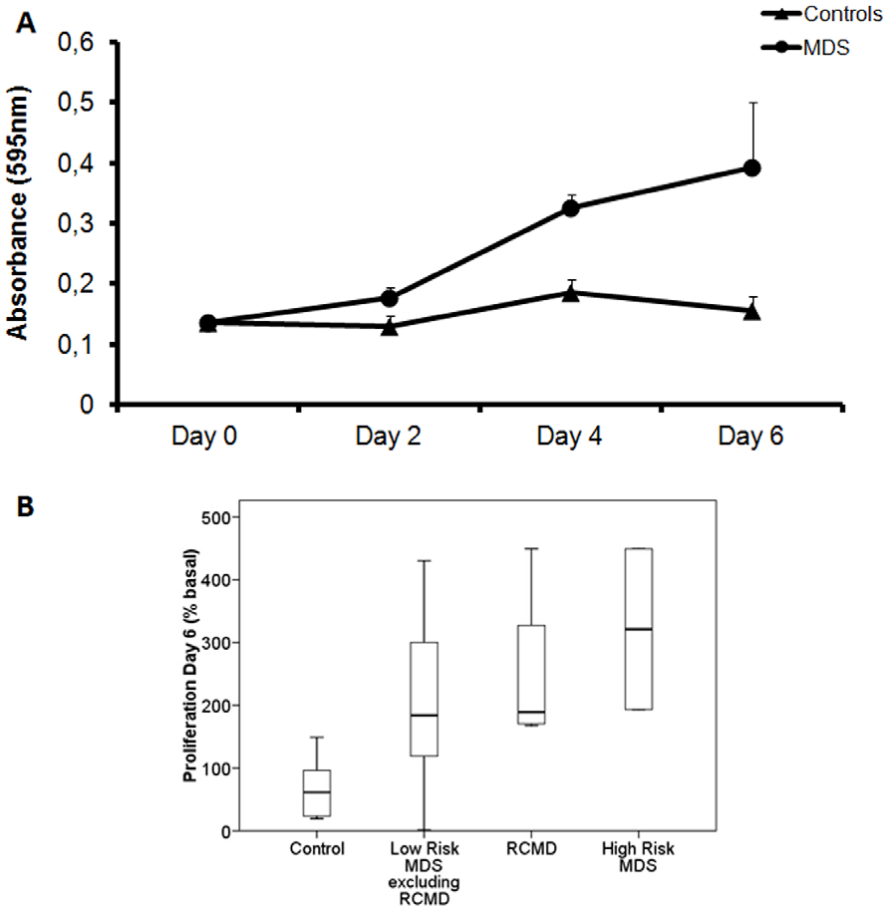
Effect of MDS BM microenvironment on BMEC-1 proliferation. (A) BMEC-1 proliferation curve. To analyze the effect of the BM supernatant fluid from MDS patients and controls on BMEC-1 proliferation, the cell line was incubated with BM supernatant fluid. The cell number was estimated by MTT at two, four or six days. The measurement of absorbance is indicative of the rate of cell proliferation and each value of each patient is the mean of four independent experiments. Each point is the mean of these values ± SEM. The graphics show the increase of proliferation in MDS patients. ANOVA test was used to analyze the overall MDS results at sixth day. The proliferation was 2.4 times higher in MDS than controls (*p*<0.005). (B) The box plot compares median levels of BMEC-1 proliferation at sixth day in the different subtypes of MDS. Whiskers represent the range. Significant differences between RCMD and the control group (*p*<0.01), the other low-risk MDS and the control group (*p*<0.05) and high-risk MDS patients and the controls (*p*<0.05) were observed by Mann-Whitney test. MDS: myelodysplastic syndrome; BM: bone marrow; BMEC-1: bone marrow endothelial cells; MTT: Thiazolyl Blue Tetrazolium Bromide; SEM: standard error of the mean; RCMD: refractory cytopenia with multilineage dysplasia. (Controls n = 8; MDS n = 14; Low-Risk MDS excluding RCMD n = 6; RCMD n = 4; High-Risk MDS n = 4).

Endothelial proliferation is crucial in the process of angiogenesis. First, we studied the effect of BM supernatant fluid from MDS patients and controls on BMEC-1 proliferation, during eight days. Endothelial cells reached the peak of maximum proliferation at the sixth day (Figure 1A) and the values at this day were chosen for comparison between different subgroups.

At the sixth day, MDS BM supernatant fluid induced a significantly higher proliferation rate in BMEC-1 than the control BM supernatant fluid (*p*<0.005). Specifically, the proliferation was 2.4 times higher in MDS than controls (Figure 1A) and this significant difference (*p*<0.05) was detected in all three MDS subgroups analyzed (low-risk excluding RCMD, RCMD, high-risk) while no statistical differences in the endothelial proliferation were observed between the three groups of MDS themselves (Figure 1B).

### BM Microenvironment from MDS Patients Induces Abnormal Tube Formation

**Figure 2:**
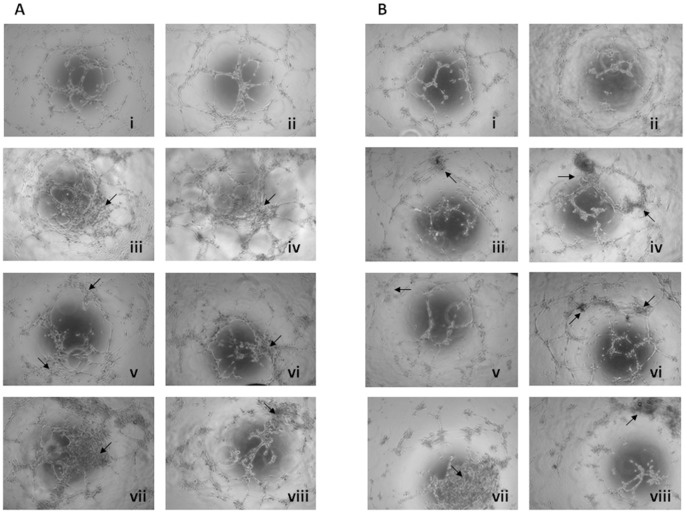
Effect of the MDS BM microenvironment on BMEC-1 and HMVEC-L tube formation. BMEC-1 (A) and HMVEC-L (B) were seeded at a concentration of 8,000 cells per well of 96-well plate and incubated for 7 h at 37°C in 5% CO_2_. The endothelial tube formation was photographed at 5 h using a phase contrast inverted microscope. Each experiment was performed in duplicate. The pictures show the appearance of endothelial cell tubes on Matrigel® precoated plates in culture medium (i) and BM supernatant fluid from healthy control (ii), RA (iii), RARS (iv), 5q syndrome (v), RAEB (high-risk MDS) (vi) and RCMD (vii-viii) patients at 1∶10 dilution in culture medium. As the arrows show in the figure, the tube morphology was strikingly influenced by BM supernatant fluid from MDS (iii-viii) with respect to the controls (ii). The tubes originated after the incubation of BMEC-1 or HMVEC-L with the BM supernatant fluid from RCMD patients (vii-viii) were almost completely disrupted and formed closed capillary networks. MDS: myelodysplastic syndrome; BM: bone marrow; BMEC-1: bone marrow endothelial cells; HMVEC-L: lung-derived normal human microvascular endothelial cells; RA: refractory anemia; RARS: refractory anemia with ring sideroblast; RAEB refractory anemia with excess of blasts; RCMD: refractory cytopenia with multilineage dysplasia. (Controls n = 13; RA n = 5; RARS n = 6; 5q syndrome n = 2; RAEB n = 4; RCMD n = 7).

Tube formation indicates the extent of angiogenesis and is considered to be an important prognostic factor in this process. To investigate the BM supernatant fluid effects on BMEC-1 (Figure 2A) and HMVEC-L (Figure 2B) tube generation, we used a common method for gauging in vitro angiogenesis, the capillary-like tube formation assay on Matrigel®. As a control, the endothelial tube formation by BMEC-1 and HMVEC-L was maintained in culture medium (Figure 2Ai and 2Bi, respectively). The tubes seem to be completely formed after five hours of incubation, and this time was used to compare the effect of different MDS BM supernatant fluid.

When the endothelial cells lines were cultured with BM supernatant fluid from the control, there was a well organized tube formation. The endothelial tube appearance in cells treated with BM supernatant from controls and cell maintained in culture medium was similar (Figure 2A and B i–ii). By contrast, the tube morphology was strikingly influenced by BM supernatant fluid from MDS patients. Therefore, BM supernatant fluid from MDS patients induces morphogenetic changes in the endothelial tube formation (Figure 2A and B iii–viii). MDS-treated BMEC-1 and HMVEC-L tented to assemble and form aggregates along the tube-like structures, which was not observed in control cells (Arrows in Figure 2). Incubation with different BM supernatant fluid from MDS stimulated the capillary network aggregation of endothelial cells, including increasing areas covered by the cells and lengths of network compared to controls (Figure 2A and B iii–viii). It should be noted that the tubes originated after the incubation of BMEC-1 and HMVEC-L with the BM supernatant fluid from RCMD patients almost completely disrupted the capillary networks (Figure 2 A and B vii–viii).

### Endoglin and Other Angiogenic Factors Expression Differences in RCMD Patients with Respect to Other MDS Patients

**Figure 3:**
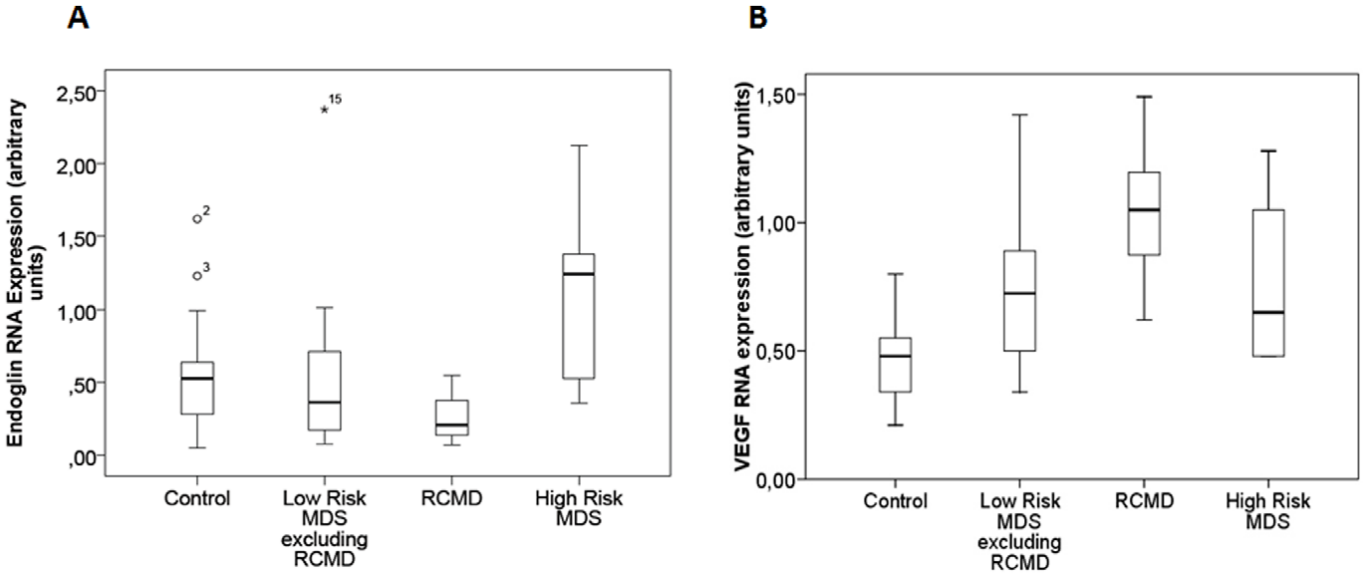
*ENG* and *VEGF* RNA expression in mononuclear BM cells of MDS subtypes. The box plot compares median of *ENG* and *VEGF* expression levels in BM mononuclear cells between the different MDS groups and controls. The gene expression levels were analyzed by RT-PCR. Each sample was performed in triplicate. Value of each patient is the mean of these three experiments. Mann-Whitney test was used to analyze the results. The box plot compares the RNA expression in BM mononuclear cells of subtypes of MDS. Whiskers represent the range. A down-regulation of *ENG* was showed in RCMD cases (*p*<0.05). By contrast, *ENG* expression in high-risk MDS patients was higher than in controls or in the other MDS (*p*<0.05). No significant differences in low-risk MDS excluding RCMD patients in *ENG* expression with respect to the healthy controls were found (A). The low-risk MDS groups showed over-expression of *VEGF* with respect to the control group (*p*<0.05). Moreover, patients with RCMD showed the highest values in the expression of this gene with respect to the other low-risk MDS. No significant differences in high-risk MDS patients in *VEGF* expression with respect to the healthy controls were found (B). ENG: endoglin; VEGF: vascular endothelial grow factor; BM: bone marrow; MDS: myelodysplastic syndrome; RCMD: refractory cytopenia with multilineage dysplasia; RAEB: refractory anaemia with excess of blasts. (Controls n = 13; Low-Risk MDS excluding RCMD n = 22; RCMD n = 12; High-Risk MDS n = 16).

Clonal-derived hematopoietic myeloid progenitor cells may facilitate the angiogenesis without directly participating in this process by promoting the activation of normal BMEC. To better understand the role of ENG in the angiogenesis of MDS patients, a gene expression study was performed. RNA obtained from BM mononuclear cells from MDS was used to analyze four angiogenic factors: *ENG*, *VEGF*, *HIF1* and *FN1*. Regarding *ENG* expression, no differences were observed between all MDS patients as a single group and the control group (Figure S1A). However, marked differences in the *ENG* levels were observed in the separate analysis of MDS groups. Thus a down-regulation of *ENG* expression was patent in RCMD patients (*p*<0.05). By contrast, *ENG* expression in high-risk MDS cases was higher than in controls (*p*<0.05). No differences were found between the *ENG* levels of low-risk MDS (excluding RCMD) and healthy controls (Figure 3A).

Overall the expression levels of *VEGF*, *HIF1* and *FN1* in MDS were significantly higher (*p*<0.05) than in controls (Figure S1B–D). Thus, the low-risk MDS groups (including RCMD) showed over-expression of *VEGF* (Figure 3B), *HIF* and *FN1* (Figure S2) with respect to the control group (*p*<0.05). Moreover, patients with RCMD showed the highest values in the expression of these three genes with respect to the other low-risk MDS (Figure 3B and Figure S2). By contrast, no differences in high-risk MDS patients regarding *VEGF*, *HIF1* and *FN1* expression with respect to the control group were observed (Figure 3B and Figure S2).

### Patients with RCMD Display High Concentrations of Anti-angiogenic Soluble Factors in the BM Microenvironment

To assess the levels of angiogenic and anti-angiogenic factors present in the BM supernatant fluid in different MDS groups, ELISA assays were carried out in the BM supernatant fluid from MDS patients and controls. Therefore, circulating levels of sENG and sFLT-1 as well as VEGF were analyzed.

**Figure 4:**
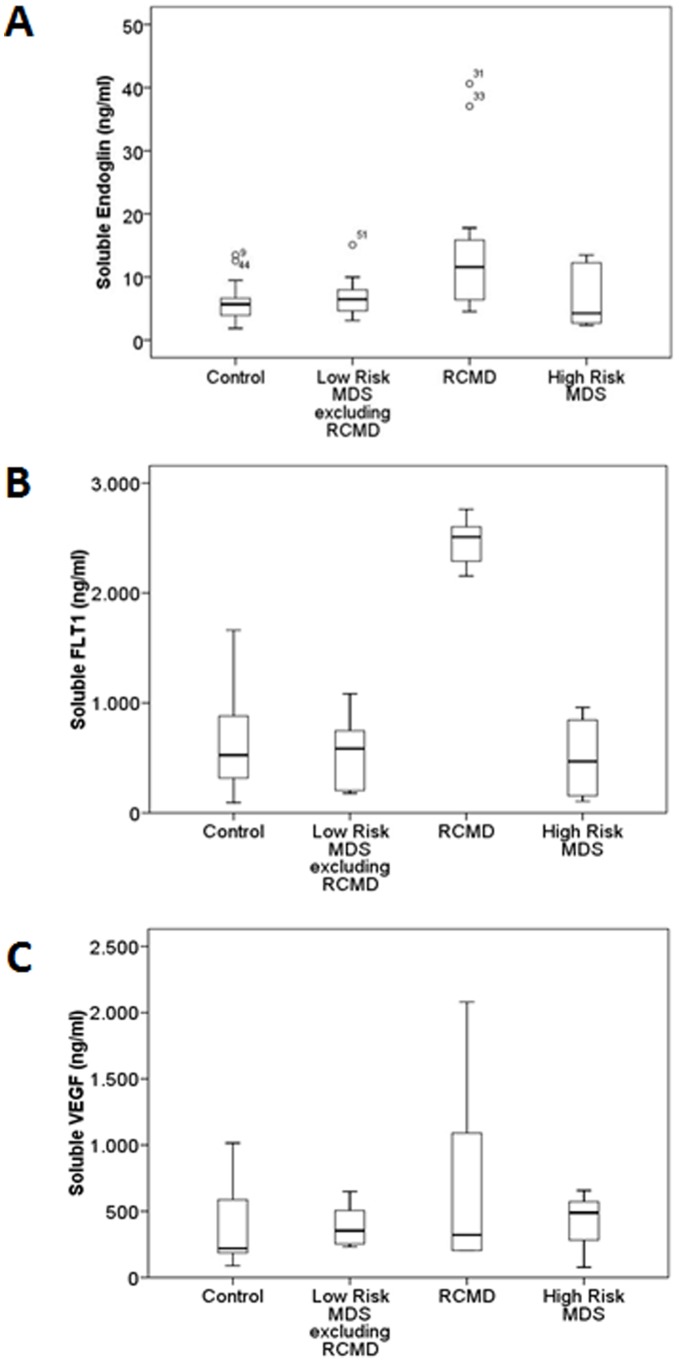
Soluble angiogenic factors in MDS BM microenvironment. The box plot compares median levels of sENG, sFLT-1 and sVEGF in BM supernatant fluid of various types of MDS. To measure the levels of angiogenic factors present in the BM supernatant fluid in the different MDS groups, ELISA assays were carried out in the BM supernatant fluid from MDS patients and controls. Whiskers represent the range. Mann-Whitney test showed that sENG concentrations in BM supernatants was higher in RCMD with respect to the healthy cases (*p*<0.005), the remaining low-risk MDS (*p*<0.05) and high-risk patients (*p* = 0.05) (A). RCDM displayed higher levels of sFLT-1 with respect to the controls (*p* = 0.001), the remaining low-risk MDS (*p*<0.005) and the high-risk MDS patients (*p*<0.005) (B). No significant differences in sVEGF concentration of MDS groups were found (C). MDS: myelodysplastic syndrome; BM: bone marrow; ENG: endoglin; sFLT-1: fms-like tyrosine kinase 1; VEGF: vascular endothelial grow factor; RCMD: refractory cytopenia with multilineage dysplasia. (Controls n = 24; Low-Risk MDS excluding RCMD n = 15; RCMD n = 15; High-Risk MDS n = 6).

The Figure 4 summarizes the results: RCDM displayed higher levels of sENG with respect to the controls (*p*<0.005), the remaining low-risk MDS (*p*<0.05) and the high-risk MDS patients (*p* = 0.05). Moreover, sFLT-1 concentrations in BM supernatants were higher in RCMD with respect to the healthy cases (*p* = 0.001), the remaining low-risk MDS and high-risk patients (*p*<0.005) (Figure 4B). By contrast, the study lacked in detect differences in the concentration of VEGF in the three MDS groups analyzed (Figure 4C).

## Discussion

Myelodysplastic syndromes (MDS) are clonal stem cell diseases in which altered angiogenic mechanisms have been described. In the present study, a combined analysis of gene expression, angiogenesis-related soluble factors and functional angiogenesis-related studies were carried out in bone marrow (BM) of patients with MDS. The results demonstrated marked differences in angiogenesis in the subtypes of MDS. Thus, the patients with refractory cytopenia with multilineage dysplasia (RCMD) showed an abnormal angiogenesis characterised by an increased level of soluble endoglin (sENG).

The involvement of the microenvironment in MDS disorders has been stressed. However, most of angiogenesis studies in MDS have been focused in plasma from peripheral blood, while results regarding the BM microenvironment analysis are scarce [8], [30], [31]. In the present report functional studies in the non-cellular portion of BM were performed. A proliferation assay showed that MDS BM supernatant fluid stimulated bone marrow endothelial cells (BMEC-1) proliferation more than supernatant fluid from controls. Our results support the aberrant angiogenesis in MDS previously analyzed by other techniques [26], [32], [33]. In addition, the generation of new vessels in MDS is critical in the multistep process of conversion from normal to dysplastic BM [5], [33]. The endothelial tube formation by BMEC-1 and HMVEC-L in Matrigel® was performed in the presence of BM supernatant fluid from MDS patients and differences in thickness, structure and density of the formed tubes were observed. In addition, the differences between the capillary-like structures originated by BMEC-1 in RCDM and the remaining patient groups were evident. RCMD cases showed less extensive capillary network and reduced vessel formation (Figure 2vii–viii). BM angiogenesis in MDS has been usually studied by measuring the microvascular density by immunohistochemistry [8], [26], [31], [34]. However, this is the first time, to our knowledge, that the formation of pseudocapillaries in MDS BM supernatant fluid by means of functional techniques has been carried out. Thus, the present studies demonstrated that MDS patients displayed an abnormal angiogenesis characterized by a high endothelial proliferation and aberrant pseudocapillary formation. Therefore the BM microenvironment plays an important role in this aberrant angiogenesis.

It has been demonstrated that leukemic cells may have intimate interactions with bone marrow endothelial cells (BMEC) and can elicit the sprouting of new blood vessels from pre-existing capillaries by the active release of angiogenic factors [32], [33], [35]. Based on this consideration, we focused our attention on the analysis of several molecules in BM mononuclear cells from MDS that have been reported to be involved in the angiogenesis processes and could be influencing on BMEC behavior. Thereby, we demonstrated that vascular endothelial grow factor (*VEGF*), hypoxia-inducible factor 1-alpha (*HIF*), and fibronectin (*FN1*) expression were differentially over-expressed in low- risk MDS patients, including RCMD cases. These findings are supported by some studies where *VEGF* and other angiogenic factors were significant increase in overall MDS group [34]. In addition, the over-expression of these molecules could explain the abnormal proliferation and tube formation by endothelial cell lines in low-risk MDS.

However, angiogenesis involves two stage of vascular development: the differential growth and sprouting of endothelial tubes and the remodeling the primary endothelial network into a mature circulatory system. Endoglin gene (*ENG*) encodes an endothelial transmembrane protein that is required for both processes [14], [21]. As ENG staining represents a powerful marker to quantify tumor angiogenesis [13] we have evaluated the expression of *ENG* in MDS cells and we have demonstrated an over-expression in the high-risk cases. *ENG* expression is elevated during alterations in vascular structure and has been associated to many cancers, including breast, ovary, prostate and cervical cancer [20]. As cellular *ENG* levels regulate the formation of new blood vessels [14], ENG antibodies have been successfully used to elicit anti-angiogenic effects in tumor-associated endothelium mouse models where *ENG* was highly expressed [36]. These advances will provide new approaches for the development of new therapies for high-risk MDS patients.

Interestingly, *ENG* expression was significantly lower in RCMD patients than in cell from healthy controls. This event may resemble other vascular diseases, such as the hereditary hemorrhagic telangiectasia type I (HHT). HHT patients have significantly lower ENG levels and are characterized by arteriovenous malformations and focal loss of capillaries [37]. In addition, it has been reported that isolated murine *Eng*+/− cells display impaired capillary tube formation and significantly less vascular structures compared to wild type mice [28]. Based on these findings, we suggest that the under-expression of *ENG* in RCMD patients could be associated with the decreased blood vessel formation *in vitro* models of angiogenesis observed in the same group of patients. Furthermore, the expression variations in the diverse angiogenic factors could play different roles in the MDS subtypes suggesting different mechanisms involved in the pathogenesis of these diseases leading to a different angiogenesis in patients with RCMD with respect to the other MDS patients.

The results of *ENG* gene expression led us to investigate their presence in the extracellular medium as well as the levels of soluble fms-like tyrosine kinase 1 (sFLT-1) (anti-angiogenic factor) and VEGF (angiogenic factor). RCMD patients showed the highest levels of sENG and sFLT-1 in BM supernatant fluid with respect to both the other MDS and the control group (Figure 4). By contrast, the VEGF levels were similar to the controls. A high concentration of sENG has been also described in acute myeloid leukemia and chronic myeloproliferative disorders [38] and in patients with pathologies associated to vascular dysfunction [39]. Elevated circulating concentrations of sENG and sFLT-1 have been showed in the maternal endothelial dysfunction called preeclampsia. In fact, some authors suggest that sENG may act in concert with sFLT-1 to induce severe preeclampsia [21], [40]. In addition, some studies have displayed that sFLT-1 binds to and neutralizes the pro-angiogenic actions of VEGF and the contributions of sENG and sFLT-1 to the pathogenesis of maternal preeclampsia are, at least in part, related to their inhibition of TGFβ and VEGF, respectively [41]. This finding could explain the mitigated VEGF secretion observed in RCMD patients in relation to the expression gene in the same group of patients. Based on our results, we suggest that the soluble form of ENG antagonizes the membrane bound form in RCMD patients and therefore potentiates the anti-angiogenic actions of sFLT-1, by disrupting the capacity to form capillary tubes of BMEC-1 and HMVEC-L as we have previously showed in this group of patients.

Angiogenesis is a balanced process between pro and anti-angiogenic factors. In MDS patients, our results suggest the presence of an altered balance that could be involved in RCMD patients. In fact, RCMD patients showed high expression levels of pro-angiogenic factors such as *VEGF*, *HIF*, and *FN1*. In contrast, this group of MDS had low *ENG* expression, high levels of sENG and sFLT-1 in BM microenvironment, a decrease level of VEGF with respect to the expression gene and the reduced vessel formation by endothelial cell lines. Regarding the increased BMEC-1 proliferation observed in RCMD there are conflicting evidences: endothelial cell proliferation is key early event in angiogenesis, but some studies have demonstrated that myeloid malignancies with high levels of sENG are characterized by a high cellular proliferation rate in BMEC and even, in myelopoietic lineage what could explain the high proliferation in an anti-angiogenic environment [28], [38]. Therefore we suggest that the RCMD display features that tip the balance of angiogenesis and appear to be impairing this process.

Previous studies have demonstrated an abnormal angiogenesis in MDS. However most of them have analyzed the differences between the low-risk and high-risk patients while the RCDM patients were not included as an independent group [26], [34]. The RCMD has been recently proposed by the WHO classification as a specific MDS disorder [2] and the present study showed these patients had a different pattern of angiogenesis. These results provide new insights in the molecular mechanisms of RCMD patients that could be ENG-related. Furthermore, recently, it has been suggested that the inhibition of putative protease involved in sENG shedding may be of therapeutic benefit in the treatment of preeclampsia [20]. These observations could provide new therapeutic approaches for this specific subtype of MDS.

## Supporting Information

Figure S1 ***ENG*** **,** ***VEGF*** **,** ***HIF1*** **and** ***FN1*** **RNA expression in mononuclear MDS BM cells.** The box plot compares median of *ENG, VEGF*, *HIF1* and *FN1* expression levels in BM mononuclear cells between MDS and controls. To analyze the gene expression levels of the angiogenic factors we used RT-PCR. Each value of each sample is the mean of three independent experiments. The box plot shows the differences between the samples expression distributions in control and MDS group. Whiskers represent the range. Mann-Whitney test was applied in all cases. No significant differences were found in *ENG* expression between MDS patients and control group (A). Overall the expression levels of *VEGF*, *HIF1* and *FN1* in MDS were significantly higher (p<0.05) than levels of controls (B-D). ENG: endoglin; VEGF: vascular endothelial grow factor; HIF1: hypoxia-inducible factor 1-alpha; FN1: fibronectin; BM: bone marrow; MDS: myelodysplastic syndrome. (Controls n = 13; MDS n = 50).
(TIF)

Figure S2 ***HIF1*** **and** ***FN1*** **RNA expression in mononuclear BM cells of MDS subtypes.** The box plot compares median of *HIF1* and *FN1* expression levels in BM mononuclear cells between the different MDS groups and controls. The gene expression levels were analyzed by RT-PCR. Each sample was performed in triplicate. Each value of each patient is the mean of these three experiments. Mann-Whitney test was used to analyze the results. The box plot compares the RNA expression in BM mononuclear cells of subtypes of MDS. Whiskers represent the range. The low-risk MDS groups showed over-expression of *HIF1* and *FN1* with respect to the control group (p<0.05). Moreover, patients with RCMD showed the highest values in the expression of these two genes with respect to the other low-risk MDS. Overall no significant differences in high-risk MDS patients in *HIF1* and *FN1* expression with respect to the healthy controls were found. HIF1: hypoxia-inducible factor 1-alpha; FN1: fibronectin; BM: bone marrow; MDS: myelodysplastic syndrome; RCMD: refractory cytopenia with multilineage dysplasia; RAEB: refractory anaemia with excess of blasts. (Controls n = 13; Low Risk MDS excluding RCMD n = 22; RCMD n = 12; High Risk MDS n = 16).
(TIF)

Table S1 **Clinical and biological characteristics of MDS patients.**
(DOCX)
