# Supplementary figures and images for: Alteration in Endoglin-Related Angiogenesis in Refractory Cytopenia with Multilineage Dysplasia
Source: PLoS One. 2013 Jan 16;8(1):e53624. doi: 10.1371/journal.pone.0053624 (PMC3547003; doi:10.1371/journal.pone.0053624)

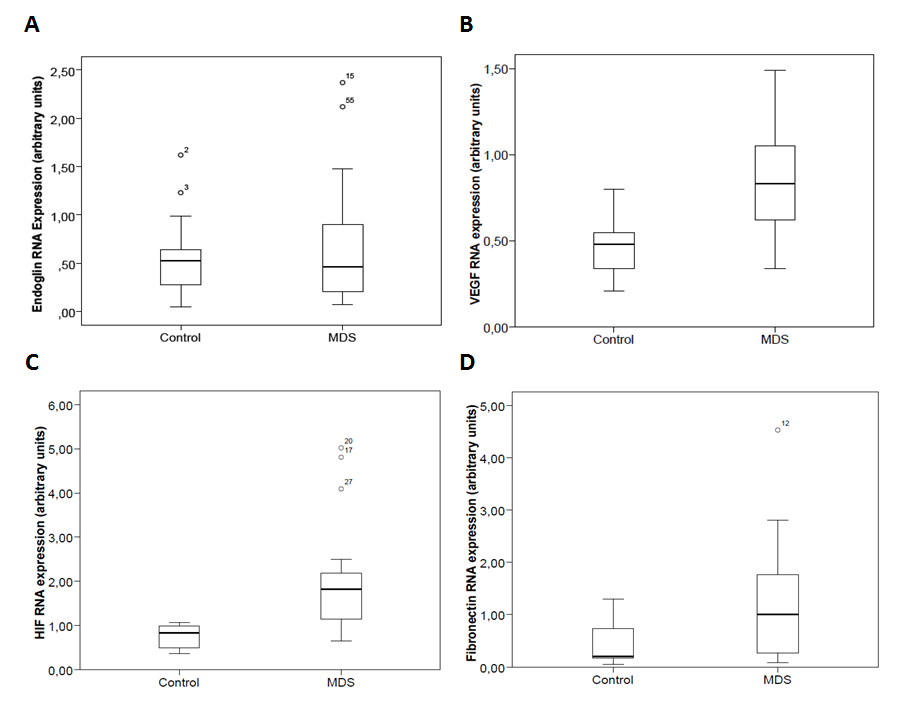

Supplement: Figure S1 — ENG , VEGF , HIF1 and FN1 RNA expression in mononuclear MDS BM cells. The box plot compares median of ENG, VEGF, HIF1 and FN1 expression levels in BM mononuclear cells between MDS and controls. To analyze the gene expression levels of the angiogenic factors we used RT-PCR. Each value of each sample is the mean of three independent experiments. The box plot shows the differences between the samples expression distributions in control and MDS group. Whiskers represent the range. Mann-Whitney test was applied in all cases. No significant differences were found in ENG expression between MDS patients and control group (A). Overall the expression levels of VEGF, HIF1 and FN1 in MDS were significantly higher (p<0.05) than levels of controls (B-D). ENG: endoglin; VEGF: vascular endothelial grow factor; HIF1: hypoxia-inducible factor 1-alpha; FN1: fibronectin; BM: bone marrow; MDS: myelodysplastic syndrome. (Controls n = 13; MDS n = 50). (TIF) [file pone.0053624.s001.tif]

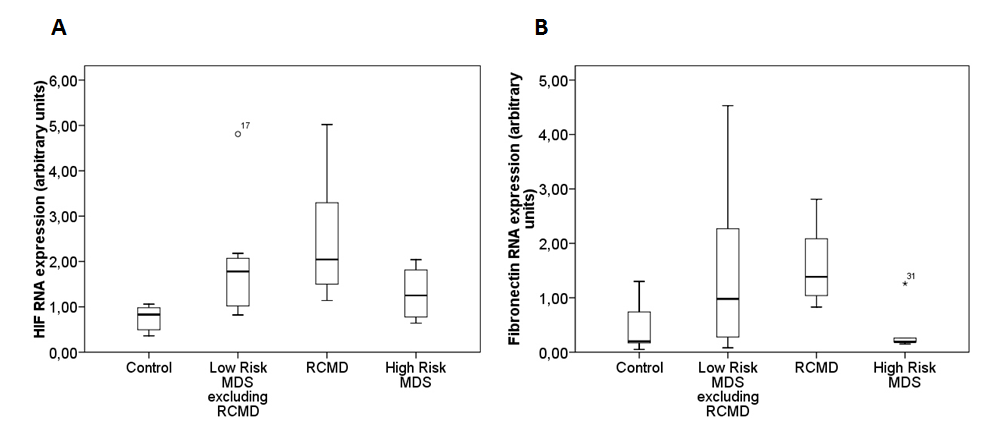

Supplement: Figure S2 — HIF1 and FN1 RNA expression in mononuclear BM cells of MDS subtypes. The box plot compares median of HIF1 and FN1 expression levels in BM mononuclear cells between the different MDS groups and controls. The gene expression levels were analyzed by RT-PCR. Each sample was performed in triplicate. Each value of each patient is the mean of these three experiments. Mann-Whitney test was used to analyze the results. The box plot compares the RNA expression in BM mononuclear cells of subtypes of MDS. Whiskers represent the range. The low-risk MDS groups showed over-expression of HIF1 and FN1 with respect to the control group (p<0.05). Moreover, patients with RCMD showed the highest values in the expression of these two genes with respect to the other low-risk MDS. Overall no significant differences in high-risk MDS patients in HIF1 and FN1 expression with respect to the healthy controls were found. HIF1: hypoxia-inducible factor 1-alpha; FN1: fibronectin; BM: bone marrow; MDS: myelodysplastic syndrome; RCMD: refractory cytopenia with multilineage dysplasia; RAEB: refractory anaemia with excess of blasts. (Controls n = 13; Low Risk MDS excluding RCMD n = 22; RCMD n = 12; High Risk MDS n = 16). (TIF) [file pone.0053624.s002.tif]
