# Supplementary material for: Alteration in Endoglin-Related Angiogenesis in Refractory Cytopenia with Multilineage Dysplasia
Source: PLoS One. 2013 Jan 16;8(1):e53624. doi: 10.1371/journal.pone.0053624 (PMC3547003; doi:10.1371/journal.pone.0053624)
Supplement: Table S1 — Clinical and biological characteristics of MDS patients. (DOCX) [file pone.0053624.s003.docx]

| **Table S1. Clinical and biological characteristics of MDS patients** | | |
| --- | --- | --- |
|  |  |  |
| MDS subtype | RCMD | 29.9% |
|  | RA | 19.6% |
|  | RARS | 22.7% |
|  | 5q- Syndrome | 5.1% |
|  | RAEB | 22.7% |
| Gender | Female | 34.1% |
|  | Male | 65.9% |
| Age (range) | | 75 (21-92) |
| BM blast (range) | | 1.5 (0-20) |
| Hemoglobin (g/dl) (range) | | 9.8 (4.7-18.4) |
| White blood cells/mm^3^ (range) | | 2500 (100-15200) |
| Platelets/mm^3^ (range) | | 172500 (7000-823000) |
| Karyotype | No mitosis | 12.1% |
|  | Normal | 64.8% |
|  | One alteration | 17.6% |
|  | Two or more alterations | 5.5% |
|  |  |  |
| ^1^Results expressed as median or percentages. | | |
